# A deep learning pipeline for automatic analysis of multi-scan cardiovascular magnetic resonance

**DOI:** 10.1186/s12968-020-00695-z

**Published:** 2021-04-26

**Authors:** Hakim Fadil, John J. Totman, Derek J. Hausenloy, Hee-Hwa Ho, Prabath Joseph, Adrian Fatt-Hoe Low, A. Mark Richards, Mark Y. Chan, Stephanie Marchesseau

**Affiliations:** 1 Centre for Translational MR Research (TMR), National University of Singapore, Singapore, 117549 Singapore; 2 Cardiovascular & Metabolic Disorders Program, Duke-National University of Singapore Medical School, Singapore, 169857 Singapore; 3 National Heart Research Institute Singapore, National Heart Centre, Singapore, Singapore; 4 Department of Medicine, Yong Loo Lin SoM, National University of Singapore, Singapore, 117597 Singapore; 5 The Hatter Cardiovascular Institute, University College London, London, UK; 6 Cardiovascular Research Center, College of Medical and Health Sciences, Asia University, Taichung, Taiwan; 7 Tan Tock Seng Hospital, Singapore, 308433 Singapore; 8 National University Heart Centre, Singapore, 119074 Singapore; 9 Cardiovascular Research Institute, National University of Singapore, Singapore, 119228 Singapore; 10 Christchurch Heart Institute, University of Otago, 8140 Christchurch, New Zealand

**Keywords:** T1 mapping, T2 mapping, Cine short-axis, Aortic flow, Deep learning, Segmentation, Automatic analysis

## Abstract

**Background:**

Cardiovascular magnetic resonance (CMR) sequences are commonly used to obtain a complete description of the function and structure of the heart, provided that accurate measurements are extracted from images. New methods of extraction of information are being developed, among them, deep neural networks are powerful tools that showed the ability to perform fast and accurate segmentation. Iq1n order to reduce the time spent by reading physicians to process data and minimize intra- and inter-observer variability, we propose a fully automatic multi-scan CMR image analysis pipeline.

**Methods:**

Sequence specific U-Net 2D models were trained to perform the segmentation of the left ventricle (LV), right ventricle (RV) and aorta in cine short-axis, late gadolinium enhancement (LGE), native T1 map, post-contrast T1, native T2 map and aortic flow sequences depending on the need. The models were trained and tested on a set of data manually segmented by experts using semi-automatic and manual tools. A set of parameters were computed from the resulting segmentations such as the left ventricular and right ventricular ejection fraction (EF), LGE scar percentage, the mean T1, T1 post, T2 values within the myocardium, and aortic flow. The Dice similarity coefficient, Hausdorff distance, mean surface distance, and Pearson correlation coefficient R were used to assess and compare the results of the U-Net based pipeline with intra-observer variability. Additionally, the pipeline was validated on two clinical studies.

**Results:**

The sequence specific U-Net 2D models trained achieved fast (≤ 0.2 s/image on GPU) and precise segmentation over all the targeted region of interest with high Dice scores (= 0.91 for LV, = 0.92 for RV, = 0.93 for Aorta in average) comparable to intra-observer Dice scores (= 0.86 for LV, = 0.87 for RV, = 0.95 for aorta flow in average). The automatically and manually computed parameters were highly correlated (R = 0.91 in average) showing results superior to the intra-observer variability (R = 0.85 in average) for every sequence presented here.

**Conclusion:**

The proposed pipeline allows for fast and robust analysis of large CMR studies while guaranteeing reproducibility, hence potentially improving patient’s diagnosis as well as clinical studies outcome.

## Background

Cardiovascular magnetic resonance (CMR) remains an active field of innovation with new sequences being developed to enrich the obtained measurements, or extracted information from the images. In an 1-h CMR scan, a complete description of the function and structure of the heart can now be obtained, provided that accurate measurements can be extracted from the images. Computer science advances, and most specifically in artificial intelligence, have begun to impact medical practice [1] by offering high quality results that, combined with physician’s expertise, will augment diagnostic performance. In medical imaging, deep learning techniques have already shown promising results in applications such as segmentation, registration [2] and cancer detection [3].

Yet, cardiac image segmentation is a challenging task for several reasons: (i) the acquisition requires the patient’s cooperation (breath-holding instructions are given repeatedly); (ii) image reconstruction is impacted by the cardiac rhythm or lack of rhythm; (iii) the blood flow surrounding the myocardium (which often creates image artifacts); (iv) high heterogeneity in the image due to standard acquisition made of many short-axis slices. Hence, segmentation is prone to observer-variability [4], especially in the contouring of the myocardium and the right ventricle (RV). This reproducibility issue, combined with the fact that current delineation methods are extremely time-consuming, makes the development of fast, robust, accurate and clinician-friendly tools a crucial element in improving clinician productivity and patient care.

We therefore sought to develop a fully automatic multi-scan cardiac analysis pipeline. This pipeline is heavily reliant on deep learning tools, recently proposed in the medical image analysis field, to automatically segment the myocardium in CMR sequences. Using the result of the segmentation, a large set of parameters is then extracted to assess the overall cardiac condition of the patient. This pipeline can either be used alone to automatically process large cohort studies; or in conjunction with our in-house cardiac analysis software for a case per case evaluation.

## Methods

To obtain a comprehensive evaluation of the patient’s cardiac condition, several sequences are usually acquired in an hour-long CMR scan: functional to evaluate the cardiac beating process, structural to evaluate the muscle cells content and architecture; and hemodynamic to evaluate the blood flow process.

### Proposed fully automatic multi-scan analysis pipeline

Encouraged by the accuracy obtained in deep learning segmentation for cine CMR at the Automated Cardiac Diagnosis Challenge presented at STACOM workshop in 2017 [5], we intended to automatize all segmentation processes using convolutional neural networks (CNN). We chose in this pipeline to use the U-Net architecture [6] made of a series of symmetric down-sampling convolutional layers (encoding), followed by up-sampling layers and skip connections corresponding to each encoding resolution (decoding layers), which guarantees a segmentation mask of the same resolution of the initial image. To train and validate our models, we relied on several datasets composed of several health conditions (myocardial infarction, degenerative mitral valve regurgitation, cardiomyopathy and healthy subjects); and acquired on several scanners (Siemens Healthineers 3 T mMR, Trio, and Prisma; Siemens Healthineers 1.5 T Aera; and Philips Healthcare 1.5 T Achieva and Ingenia).

### Training/validation sets and available data

In order to train the models and assess of the quality of the results, the datasets available for every sequence was divided in training and validation sets in an 80:20 ratio. The cine dataset was composed of 116 subjects, representing 30,730 2D images where both the left ventricle (LV) and RV were segmented semi-automatically using our in-house software. Short-axis cine image size was typically around 208 × 256 pixels of 1.33 mm × 1.33 mm and the full stack made of 25 frames to cover the full cardiac cycle and 10–12 slices of 10 mm thickness to cover the full myocardium. The LGE data set consists of 367 patients, where the LV was manually segmented, and the scar was extracted from the myocardium either manually, or using semi-automatically Otsu thresholding, both ways being recognized previously as reliable methods for scar segmentation [7]. The native T1, post-contrast T1, and T2 datasets comprised of 40 patients each, with a ground truth segmentation of the LV manually defined. Structural images were also acquired in short-axis stack of 10–12 slices of 8-10 mm thickness to cover the full myocardium, with in-plane size ranged from 156 × 192 pixels of 1.77 mm to 1.77 mm resolution to 205 × 256 pixels of 1.33 mm × 1.33 mm resolution, depending on the scanner. Finally, for the aortic flow sequence, a data set of 96 patients was used, with aorta contours semi-automatically segmented using Segment Medviso [8]. Aortic flow sequences were made of 20–30 frames of 256 × 256 pixels of 0.78 mm × 0.78 mm resolution. All segmentations were corrected, and validated manually by experts.

### Deep learning pipeline

**Fig. 1:**
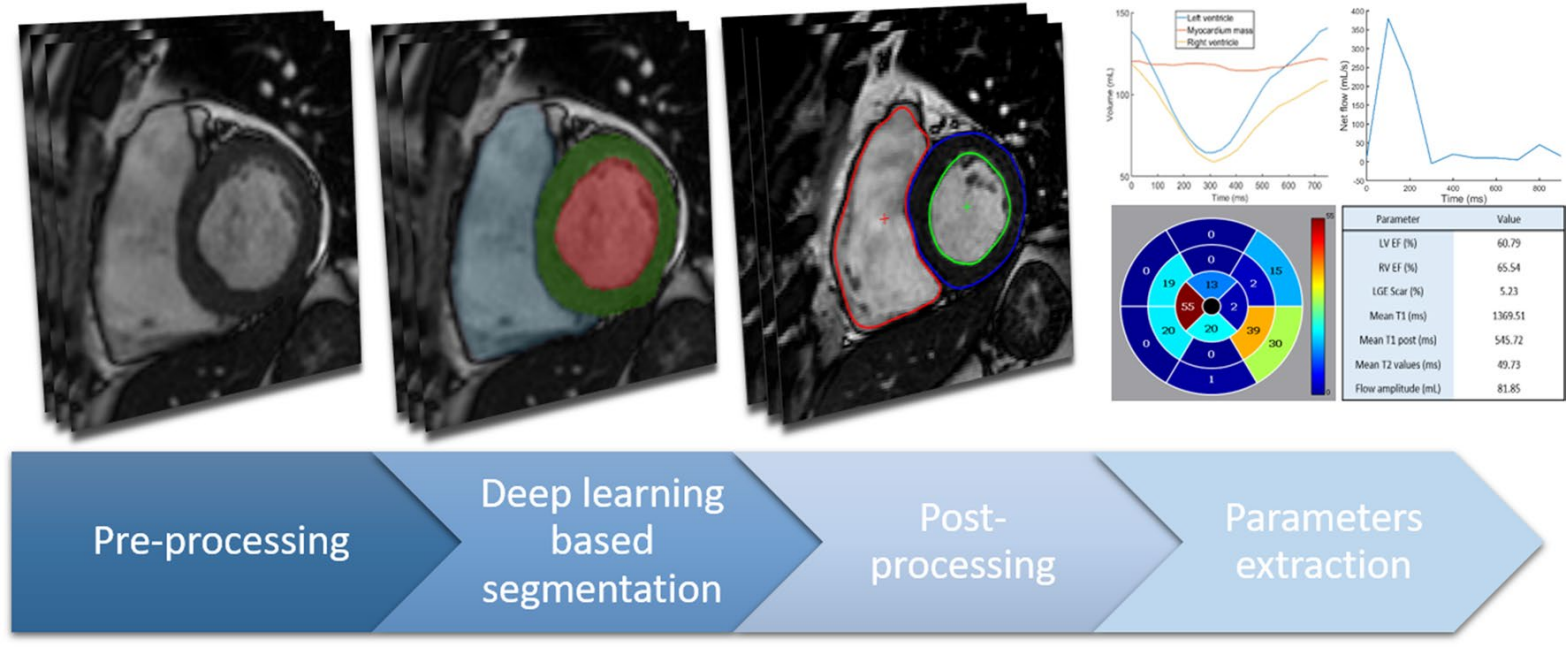
Fully automatic multi-scan CMR analysis pipeline

The proposed deep learning pipeline consists of 4 automatic steps as illustrated in Fig 1.

**Fig. 2:**
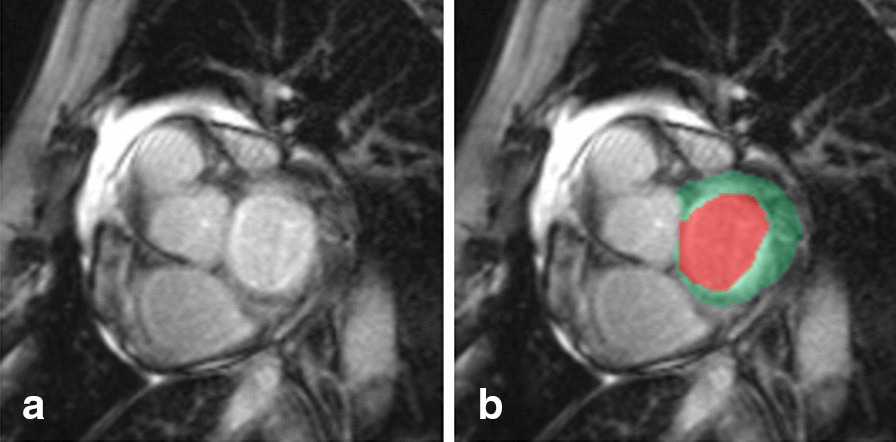
An example of over-segmentation (segmentation of an extra slice more than our ground truth) around the basal slice (**a**). **b** The blood pool and the muscle are segmented by the U-Net 2D model as the LV cavity (red) and myocardium (green). Our human experts would not segment this slice as part of the LV

1. *Pre-processing* For each sequence (cine, LGE, native T1, post-contrast T1, native T2, aortic flow), the 2D images are resized to 212 × 212 pixels of 1.37 mm × 1.37 mm resolution, with normalized intensity to deal with the possible variability in sizes, resolution and intensity.
2. *Deep learning segmentation* Each 2D image is then propagated through a sequence specific U-Net 2D model [6] that has been trained on the respective data. All models are trained to predict the anatomical structures of interest present on their sequence images. The cine, LGE, native T1, post-contrast T1, and native T2 models are trained to segment the lLV cavity, and myocardium. Additionally, the cine model is also trained to segment the RV cavity and the late gadolinium enhancement (LGE) model to predict the scar tissue in the myocardium. Similarly, the aortic flow model predicts the aorta contour. All U-Net 2D models were trained using the Adam optimizer (learning rate of 0.01, *β*_1_ = 0.9, *β*_2_ = 0.999, batch size = 5) to maximize the foreground Dice with the exception of the cine model that minimize a weighted cross entropy loss [9]. The training was performed using a GPU NVIDIA K40 for approximately 24 h.
3. *Post-processing* Finally, the U-Net 2D model generates a softmax prediction containing the probabilities of each pixel to belong to a certain region (cavity, myocardium,…). The region with the highest probability is selected for every pixel. The 2D predictions are rescaled to the original size and resolutions, and stacked to obtain a 3D mask. The largest connected component for each region is kept to remove isolated pixels. To guarantee the convexity of the LV cavity, myocardium and aorta contours, the convex hulls of their pixels are defined and chosen as the final segmentations. Moreover, in the case of the LGE, native T1, post-contrast T1, and T2, the U-Net 2D models tend to over-segment (segment an extra slice more than our ground truth) around the basal and apical slices, as shown in Fig. 2. To cope with this issue, Random Forests (RF) classifiers have been trained to identify these segmentations and discard them, improving the overall 3D segmentation. We employed Random Forests with T = 6 trees and a maximum depth of D = 6, while the features used were the mean softmax of the predicted myocardium, its mean intensity and the normalized slice position within the 3D stack. The full automatic segmentation pipeline takes less than 0.2 s for a 2D image, 50 s for a full CINE stack (≈250 images), 2 s for a stack of structural images (≈10 images), and 5 s for an aortic flow sequence (≈25 images) on a GPU (Nvidia GTX 1050). The training code and pretrained models are publicly available [10].
4. *Parameters extraction* From the segmentation of the anatomical structures, several main functional parameters are extracted depending on the analyzed sequence and clinical need. For example among others, the LV and RV ejection fraction (EF) and stroke volumes are extracted from cine images; the scar percentage within the myocardium is measured from LGE; from the native T1, post-contrast T1 and T2 maps, the mean relaxation time within the myocardium is obtained; finally, the net and backward flow amplitudes are retrieved from the segmentation of the aortic flow phase-contrast image.
5. *Full study automatic reporting* Finally, the parameters extracted for each patient in the study are reported in a large statistical file where outliers are automatically extracted using expected physiological ranges. These outliers are further processed by clinicians to evaluate the quality of the automatic segmentation and to correct them if necessary. Using a subset of the dataset, mean errors can also be measured by comparing the automatic to the corrected segmentation, as an indicator of the global confidence in the automatic measurements.

### Statistical analysis

The pipeline results on the training and validation datasets are evaluated by comparing segmentations obtained automatically against the ground truth segmentation (expert segmentation) in several ways. First, we measured the segmentation Dice score (which quantifies the amount of overlap between two segmentations and should be close to 1 for a perfect accuracy), the Hausdorff distance (HD) (which measures the local maximum distance between the two segmentations) and the mean surface distance (MSD) (which evaluates the average distance between the segmentations). The geometrical metrics were calculated in 3D following the same calculation method as in the ACDC challenge [5]. Second, we calculated the Pearson’s correlations between the physiological parameters obtained using these two segmentations; and finally we measured the mean errors between these two sets of physiological parameters. The errors were estimated by calculating the absolute difference on percentage-based parameters (such as EF, and LGE percentage), and the absolute relative difference on numerical parameters. Additionally, the intra-observer Dice and parameter reproducibility for each sequence has been estimated on a data set of 20 scans which was processed twice by the same observer within 6 months. This variability will serve as a human error.

The parameters considered are chosen among the ones usually extracted by clinicians during regular CMR study: the LV and RV EF for the Cine; the scar percentage for LGE; the mean native T1, post-contrast T1 and T2 values within the myocardium; and the net flow amplitude inside the aorta.

Overall, as can be seen in Table 1, and Table 2, the results show high robustness and compare favorably to the manual error. Furthermore, the Cine model was evaluated on the ACDC challenge [8] test dataset to compare with the state-of-the-art, and the whole pipeline was tested on two additional clinical studies to further demonstrate its robustness.

**Table 1:** Segmentation accuracy and intra-observer reproducibility for the cardiovascular magnetic resonance (CMR) sequences and anatomical structures considered

CMR sequence	Anatomical structure	Deep learning						Human performance		
		Train			Validation					
		Dice	HD (mm)	MSD (mm)	Dice	HD (mm)	MSD (mm)	Dice	HD (mm)	MSD (mm)
Cine	LV cavity	0.98 (0.01)	5.79 (2.16)	0.41 (0.12)	0.97 (0.03)	8.03 (5.47)	0.57 (0.55)	0.90 (0.01)	10.04 (1.51)	1.68 (0.19)
	LV myocardium	0.94 (0.01)	8.36 (2.22)	0.65 (0.12)	0.93 (0.04)	9.69 (2.28)	0.77 (0.43)	0.77 (0.03)	12.92 (1.84)	2.03 (0.18)
	RV cavity	0.95 (0.02)	8.99 (2.17)	0.85 (0.20)	0.92 (0.07)	10.39 (2.76)	0.95 (0.54)	0.87 (0.05)	6.59 (2.98)	1.88 (0.89)
LGE	LV cavity	0.92 (0.05)	9.91 (6.19)	1.64 (1.60)	0.90 (0.06)	11.42 (5.90)	1.74 (1.55)	0.91 (0.03)	8.84 (4.95)	1.37 (0.23)
	LV myocardium	0.86 (0.04)	12.36 (5.74)	0.89 (0.67)	0.83 (0.04)	13.24 (4.83)	1.07 (0.65)	0.83 (0.04)	12.00 (5.23)	1.40 (0.21)
	Scar	0.72 (0.13)	26.97 (9.95)	1.73 (4.09)	0.57 (0.18)	28.78 (8.41)	2.57 (2.04)	0.58 (0.11)	37.76 (11.95)	3.24 (1.89)
Native T1	LV cavity	0.96 (0.05)	6.53 (9.76)	0.86 (0.71)	0.94 (0.05)	7.34 (5.58)	1.04 (1.34)	0.88 (0.03)	11.56 (4.09)	1.99 (0.39)
	LV myocardium	0.88 (0.07)	9.09 (8.33)	0.73 (0.45)	0.88 (0.08)	12.19 (3.47)	1.09 (1.55)	0.72 (0.03)	15.61 (4.53)	1.61 (0.23)
Post-contrast T1	LV cavity	0.93 (0.05)	10.05 (7.89)	0.75 (0.58)	0.96 (0.04)	7.25 (5.54)	0.40 (0.05)	0.95 (0.02)	5.67 (2.80)	0.99 (0.34)
	LV myocardium	0.87 (0.09)	12.72 (7.49)	0.80 (0.67)	0.92 (0.03)	9.97 (4.51)	0.44 (0.09)	0.83 (0.05)	9.03 (5.33)	0.89 (0.22)
Native T2	LV cavity	0.96 (0.04)	6.16 (5.54)	0.67 (0.36)	0.96 (0.03)	5.87 (4.12)	0.49 (0.23)	0.96 (0.02)	5.82 (3.23)	0.90 (0.14)
	LV myocardium	0.89 (0.07)	9.61 (4.73)	0.66 (0.32)	0.91 (0.05)	10.96 (3.54)	0.56 (0.21)	0.86 (0.02)	7.62 (3.74)	0.89 (0.16)
Flow	Aorta	0.95 (0.01)	2.34 (0.43)	0.89 (0.16)	0.93 (0.04)	3.02 (0.89)	1.16 (0.44)	0.95 (0.02)	1.86 (0.56)	0.63 (0.29)

The mean and standard deviation (in parenthesis) are reported for the Dice metric, Hausdorff distance (HD) and mean surface distance (MSD)

*LV* left ventricle, *RV* right ventricle

**Table 2:** Correlation (R) and mean error for parameters extracted from segmentations, and intra-observer parameter reproducibility

Parameter	Deep learning				Human performance	
	Train		Validation			
	R	Error (%)	R	Error (%)	R	Error (%)
Cine LVEF (%)	0.99	1.00	0.99	1.10	0.93	3.79
Cine RVEF (%)	0.84	4.12	0.76	4.05	0.70	5.77
LGE Scar (%)	0.91	3.69	0.78	5.39	0.60	5.97
Native T1 mean values (ms)	0.99	0.69	0.98	1.00	0.83	3.24
Post-contrast T1 mean values (ms)	0.99	1.22	0.99	0.73	0.99	1.10
Native T2 mean values (ms)	0.99	0.95	0.99	0.65	0.99	0.89
AO Flow amplitude (mL)	0.99	3.16	0.89	5.77	0.92	6.03

The mean errors for the left ventricular ejection fraction (LVEF), the right ventricular ejection fraction (RVEF), and the scar represent absolute errors, while the rest are relative errors

LGE image segmentation

### Evaluation on clinical studies

The presented deep learning segmentation pipeline was used in two independent clinical studies with data acquired at various sites.

In study A, 30 patients with asymptomatic mitral regurgitation and 10 healthy subjects were recruited for a single CMR scan (cine, T1, and post-contrast T1) over the course of 6 months. Cine, native T1 and post-contrast T1 data were acquired on a 3 T CMR scanner (Trio, Siemens Healthineers), with different image resolution varying from 218 × 256 pixels of 1.4 mm × 1.4 mm to 232 × 256 pixels of 1.25 mm × 1.25 mm. The CMR scans were automatically processed using the deep learning pipeline, and all 40 cases were analyzed a second time with manual corrections. Confidence in the parameters was measured using the two sets of analysis, in terms of Pearson’s correlation, and mean absolute difference.

In study B, 95 patients were recruited in 2008 over the course of 10 years, 3 days after a primary angioplasty to undergo a baseline CMR and a 12 week follow up CMR scan, representing a total of 182 scans. Cine, LGE images were acquired on a 1.5 T CMR scanner (Symphony, Siemens Healthineers) leading to a resolution varying from 192 × 162 pixels of 1.87 mm × 1.87 mm to 256 × 208 pixels of 1.4 mm × 1.4 mm. After applying the deep learning pipeline, parameters were automatically extracted and a subset of cases (20 for cine and 15 for LGE) were selected for manual corrections based on physiological parameters out of range. Confidence in cine and LGE parameters could, therefore, be computed in terms of Pearson’s correlation and mean error to evaluate the pipeline and report on the initial research hypothesis.

## Results

### Cine image segmentation

**Fig. 3:**
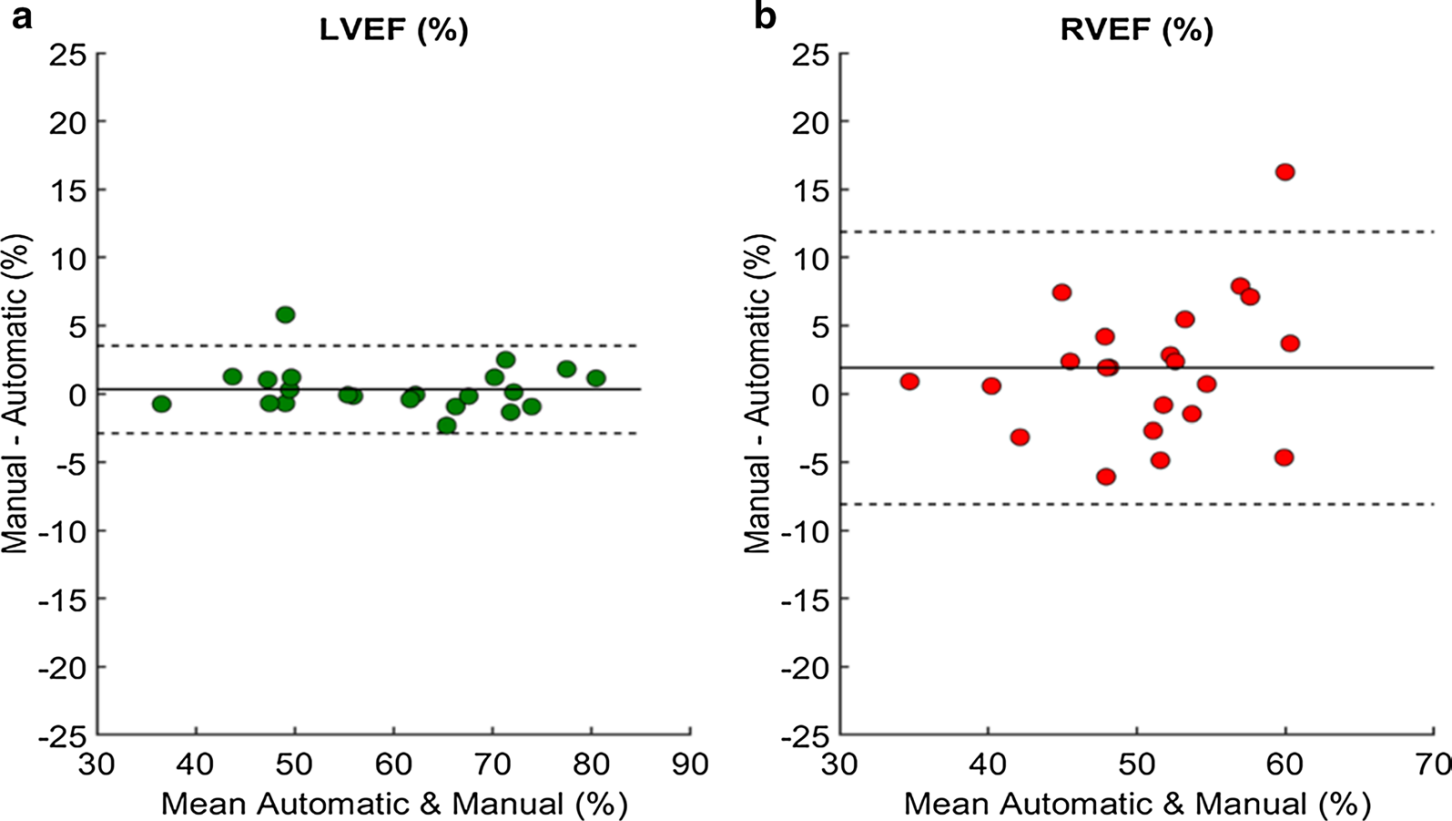
Bland–Altman plots of left ventricular ejection fraction (LVEF) (**a**) and right ventricular ejection fraction (RVEF) (**b**) measures between automated and manual measurements. The middle line denotes the mean difference (bias) and the two dashed lines denote ± 1*.*96 standard deviations from the mean. The plot **a** shows a mean difference of 0.4% with 95% limits of agreement being from -2.7% to 3.5% for LVEF. The plot **b** shows a mean difference of 1.9% with 95% limits of agreement being from -8% to 11.8% for RVEF

**Fig. 4:**
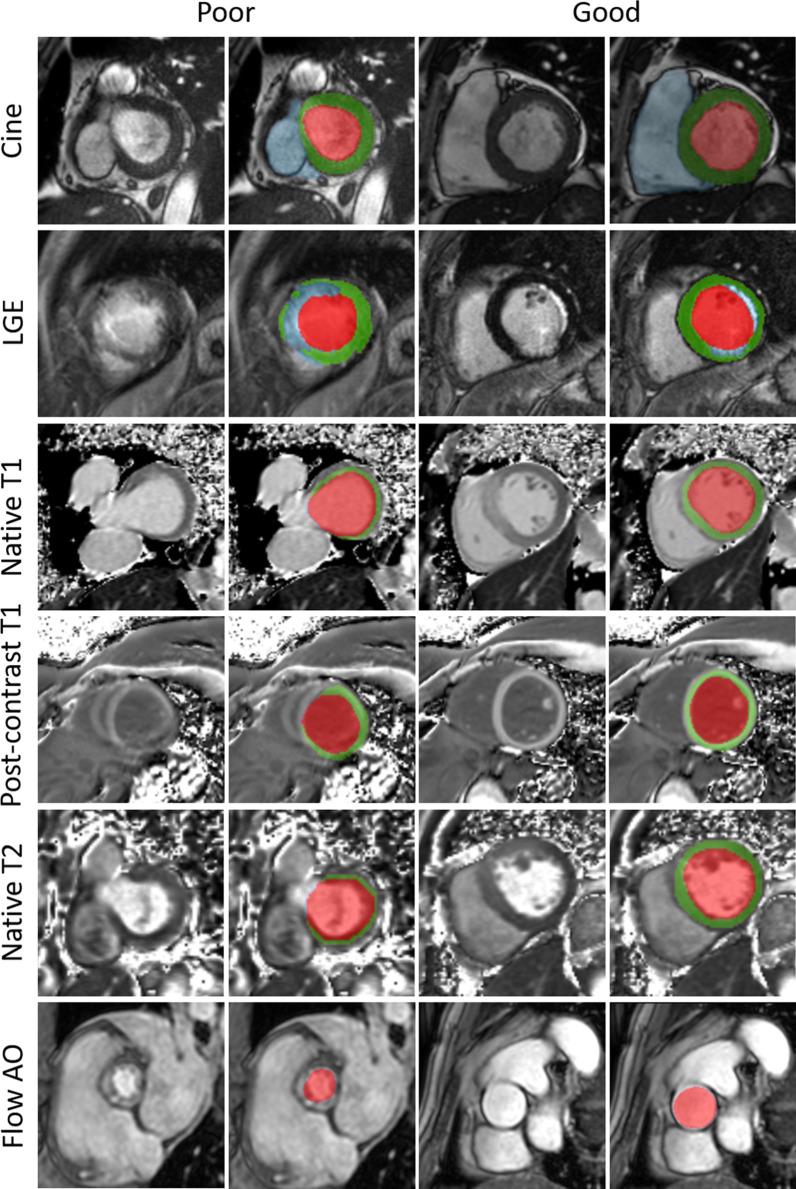
Results example of left cavity (red), left myocardium (green), right cavity (blue), scar tissue (blue), and aorta (red) segmentations obtained using the pipeline. Good, and poor results are shown for cine, LGE, T1, post-contrast T1, T2, and aortic flow images. The pipeline faces multiple challenges: the basal slice with its variability, and noise (CINE, T1, and T2); clouded and undefined boundaries (the myocardium on LGE images); artifacts compromising the shape of anatomical structures (post-contrast T1); and irregularities. Flow AO = aortic flow

As shown in Table 1, the cine model achieved very high results on both the LV (Dice = 0.97 cavity and 0.93 for myocardium) and RV (Dice = 0.92), better than the human intra-observer variability (best Dice = 0.90). The mean surface distance is 0.57 mm for the LV cavity, 0.77 mm for the LV myocardium and 0.95 mm for the RV cavity, all of which are smaller than the in-plane pixel spacing of 1.33 mm. The 3D Hausdorff distance ranges from 8.03 mm to 10.39 mm. Consequently, the correlations on the LVEF (R = 0.99) and RVEF (R = 0.76) are strong, see Table 2. Figure 3a, b show the Bland–Altman plots of the LVEF and RVEF measures using the automatic method in relation to manual segmentation. The plots show that most cases have a difference ≤ 5%. The RVEF correlation is lower than the LVEF’s for several reasons. First, the model sometimes fails to segment properly the RV at the basal slice which impacts the volume at the end-diastolic and end-systolic frames, as can be seen in Fig. 4. At the basal slice, the poor contouring is mainly due to the variability of the shape of the RV and to the noise created by the motion of the RV. This is expected as previous studies have highlighted the difficulty in manual delineation of RV contours [4] resulting in poor reproducibility (here R = 0.7). Additionally, most reported results focus on pre-selected end-diastole and end-systole frames only (as performed in [5]), while this model was required to not only segment the full cardiac cycle but also to identify the end-diastolic and end-systolic frames which increases the difficulty.

### Results on ACDC test dataset

**Fig. 5:**
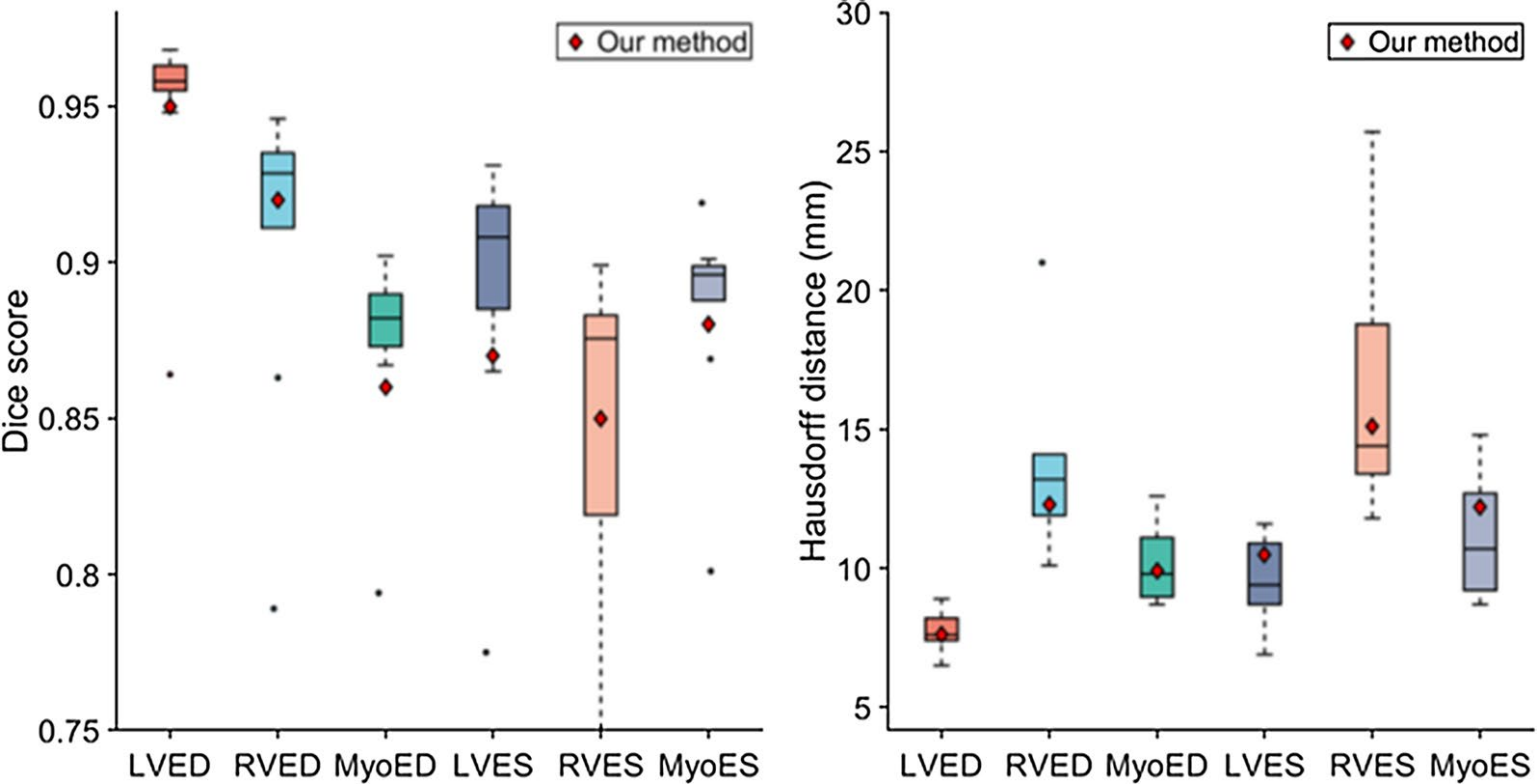
Comparison of the segmentation accuracy of our method with the top 10 methods of the ACDC challenge on the testing dataset. In these box-and-whiskers plots the middle horizontal line represents the median, box hinges represent first and third quartiles, whiskers represent extreme values within 1.5 times the interquartile range, and asterisks represent outliers. The red diamond represents the result of our method. *LV* left ventricle cavity, *RV* right ventricle cavity, *Myo* left ventricle myocardium, *ED* end-diastolic, *ES* end-systolic

**Fig. 6:**
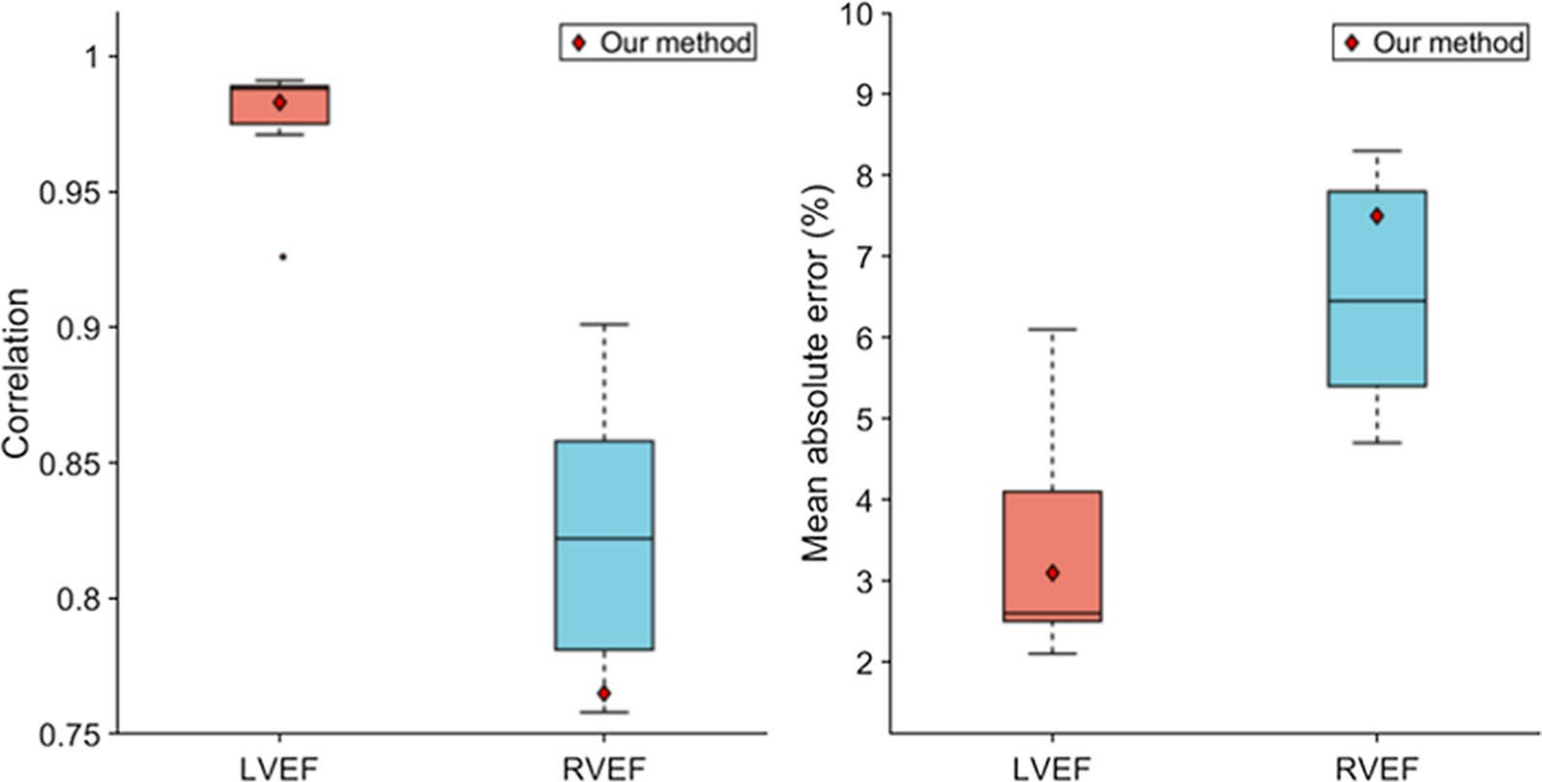
Comparison of the clinical metrics of our method with the top ten methods of the ACDC challenge on the testing dataset. In these box-and-whiskers plots the middle horizontal line represents the median, box hinges represent first and third quartiles, whiskers represent extreme values within 1.5 times the interquartile range, and asterisks represent outliers. The red diamond represents the result of our method. *LV* left ventricle, *RV* right ventricle, *EF* ejection fraction

Additionally, to compare with the methods of the ACDC challenge [5], we added to our training dataset the ACDC training dataset and retrained the cine model for 24 h. The ACDC training dataset contains 100 patients and represents about 200 2D images of end-diastolic and end-systolic frames. This dataset presents a large variability with healthy and non-healthy patients (systolic heart failure with infarction, dilated or hypertrophic cardiomyopathy, abnormal RV). After training, the cine model was tested on the ACDC test dataset containing 50 patients. In Fig. 5 and 6, the results of our method are compared with the top ten methods of the ACDC challenge. As one can see, our method is within an acceptable range for most metrics, even though the model has not been optimized specifically for the ACDC training dataset. In our case, the ACDC training dataset represents only 200 images over the ≈30,000 images used for the training. This demonstrates the ability of the model to adapt to new datasets.

### LGE image segmentation

**Fig. 7:**
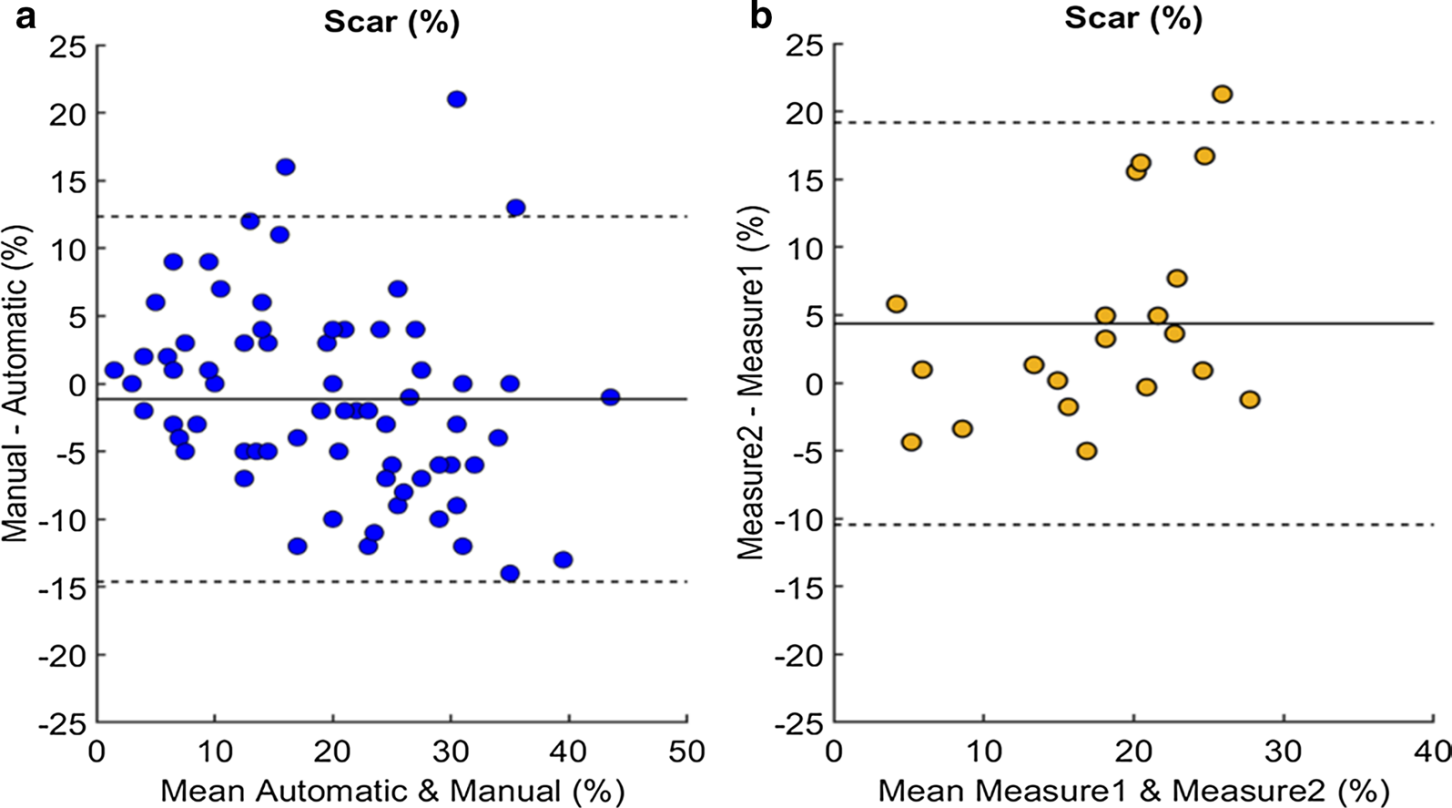
Bland–Altman plots of late gadolinium enhancement (LGE) scar percentage between automated and manual measurements (**a**), as well as between two measurements by a same human observer (**b**). The middle line denotes the mean difference (bias) and the two dashed lines denote ± 1.96 standard deviations from the mean. The plot **a** shows a mean difference of 1.1% with 95% limits of agreement being from − 14.8 to 13.5%. The plot **b** shows a mean difference of 4.4% with 95% limits of agreement being from − 10.6 to 19.4%

As shown in Table 1, the LGE model obtains satisfying results when compared to the Dice reproducibility metrics. For example, the myocardium reaches a Dice score of 0.83 with a Hausdorff distance of 13.24 mm, and a mean surface distance of 1.07 mm on the validation set. This shows that the variance and overfitting problems, originally present with our previous LGE model trained on 32 cases [11], was solved by increasing the size of the training set. The Dice score, Haussdorf distance, and mean surface distance for scar segmentation does not reach such performance despite a satisfactory correlation and mean error. This can be explained by the inability of the Dice measure, and distances to assess of the quality of the segmentation on such fragmented region of interest. However, the correlation of the scar percentage is of 0.78 with a mean error of 5.4% which remains satisfactory, see Table 2. In addition, Fig. 7 reports the Bland–Altman plots of the scar percentage using the automatic method in relation to manual segmentation, and between two manual measurements by a same human observer. The plots show a stronger bias between the two measures done by the same human observer (b) than between the automatic and manual method. Moreover, the variability of the expert segmentation, demonstrated with the human Dice score in Table 1, is in accordance with the automatic performance. Similarly to experts, the model predominantly struggles on cases where the boundaries of the myocardium, and cavity are clouded, though still providing coherent results as shown in Fig. 4.

### Native T1 map, post-contrast T1 map, and T2 map segmentation

The native T1, post-contrast T1, and T2 models offer very robust results that are superior or comparable to the intra-observer scores, as can be seen from Table 1 and Table 2. The Dices and Hausdorff distances measured range from 0.88 to 0.96 and 7.25 mm to 12.19 mm, respectively. The mean surface distances are smaller than the in-plane pixel spacing of 1.33 mm, demonstrating that the models accurately segment the LV. The parameters are highly correlated with their correlation scores around 0.99. As presented in Fig. 4 (native T1, and T2), show poorer results at the basal slice due to the variability observed within the images and among observers. Or, when dealing with artifacts that might disturb the homogeneity of the cavity and myocardium, as seen in Fig. 4 (post-contrast T1). However, these errors are negligible and do not impact the overall mean value extracted from the whole image stack.

### Random Forest over-segmentation correction

Table 3 reports the segmentation accuracy of the pipeline on the validation datasets, before and after correction by the Random Forests models. As can be seen in Table 3, the Random Forests manage to improve the overall segmentation by discarding the slices that should not have been segmented. The Dice scores of the left cavity on each CMR sequence is improved in general by more than 0.1.

**Table 3:** Comparison of the segmentation accuracy of the pipeline before and after correction by the Random Forest (RF) models

CMR sequence	Anatomical structure	Dice	
		Before RF	After RF
LGE	LV cavity	0.86 (0.06)	0.90 (0.06)
	LV myocardium	0.82 (0.05)	0.83 (0.04)
	Scar	0.54 (0.19)	0.57 (0.18)
Native T1	LV cavity	0.81 (0.08)	0.94 (0.05)
	LV myocardium	0.87 (0.08)	0.88 (0.08)
Post-contrast T1	LV cavity	0.85 (0.06)	0.96 (0.04)
	LV myocardium	0.86 (0.05)	0.92 (0.03)
Native T2	LV cavity	0.87 (0.04)	0.96 (0.03)
	LV myocardium	0.90 (0.04)	0.91 (0.05)

The mean and standard deviation (in parenthesis) of the Dice metric are reported

*LGE* late gadolinium enhancement, *LV* left ventricle

### Aortic flow image

Finally, the aorta is segmented with great precision (Dice = 0.93, HD = 0.93, MSD = 1.16). The simplicity of the geometrical shape of the aorta and its presence on all the frames enables consistently good results. Poor results are obtained when the shape of the aorta in the slice and the image intensity present irregularities as displayed in Fig. 4. However, the correlation remains very high with a value of 0.89, thus, making the model reliable.

### Evaluation on clinical studies

**Fig. 8:**
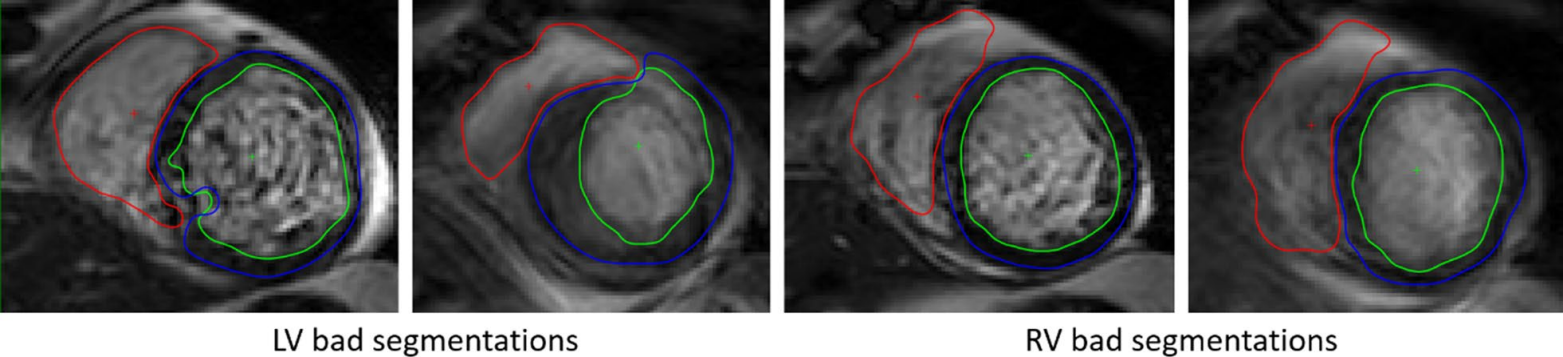
Example of bad LV (left) and RV (right) deep learning segmentations that led to an error of 5.9% in LVEF and 14% in RVEF. The poor segmentations can be explained by the poor image quality of this Cine sequence with borders very blurry or/and blood pool very inhomogeneous. *LV* left ventricle, *RV* right ventricle, *EF* ejection fraction

**Fig. 9:**
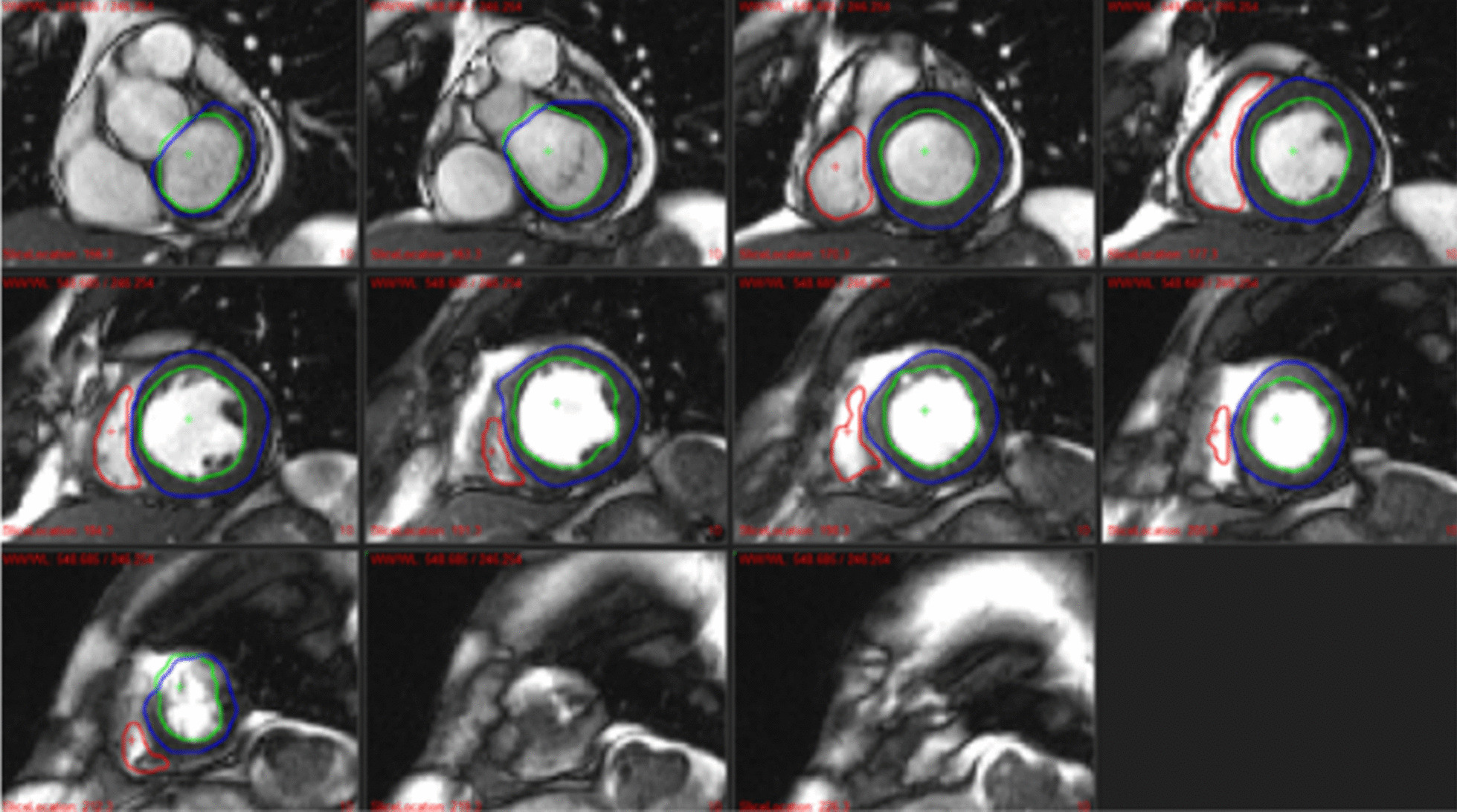
Example of deep learning segmentation for the study B case leading to the highest error in ejection fraction. The error is mainly due to over-segmentation of the basal slice for the left ventricle, and poor detection of the right ventricle for the mid to apex slices due to high contrast in fatty tissue surrounding the heart

Table 4 reports the confidence for the two studies for the key cine, LGE, T1 and post-contrast T1 parameters. It can be concluded that the corrections were not necessary to reach accurate statistical reporting of the LV parameters and could be omitted for fair results on the RV, as the errors reported are low (lower than intra-observer variability) and the correlation extremely high (≥ 0*.*87 for the LV and ≥ 0*.*73 for the RV). Scar percentage was also estimated accurately with a mean error over the 15 cases of 5.4%. As for the T1 and post-contrast T1 parameters, the automatic and manual values are highly correlated (*R* ≥ 0*.*91) with a mean absolute error of 22 ms for T1 and 10 ms for post-contrast T1. Figure 8 shows examples of worst deep learning segmentations for study A, where the important lack of precision can be explained by poor image quality. The worst automatic segmentation on study B is shown in Fig. 9, with a difference in EF of 8.3% for the LV and 9.0% for the RV.

**Table 4:** Correlation (R) and mean absolute error between the parameters extracted from the automatic and manual segmentation of the Study A and B

Parameter	Study A		Study B	
	R	Error	R	Error
LV SV (mL)	0.97	3.93	0.87	3.21
LV EF (%)	0.90	1.80	0.98	2.40
LV Mass (g)	0.99	1.86	0.99	2.14
RV SV (mL)	0.89	7.68	0.73	5.32
RV EF (%)	0.85	4.85	0.85	4.06
Scar (%)	NA	NA	0.95	5.38
Mean T1 (ms)	0.91	21.69	NA	NA
Mean post-contrast T1 (ms)	0.97	9.47	NA	NA

*EF* ejection fraction, *LV* left ventricle, *RV* right ventricle, *SV*  stroke volume

## Discussion

This paper introduces a fully automated pipeline for multi-scan CMR analysis. Our approach applies a U-net 2D model to segment the anatomical structures of interest. We demonstrated that this architecture can be used to process multiple CMR sequences (cine, LGE, native T1, post-contrast T1, native T2, and aortic flow). Our method achieved robust segmentation results that were close to identical to manual segmentation, without the limitation of intra- and inter-observer variability.

Our pipeline also compares favorably to recent methods published in the literature. For example, on cine short-axis images, we report higher accuracy than the FCN [12]: LV cavity (Dice = 0.97 vs 0.94), LV myocardium (Dice = 0.93 vs 0.88), RV cavity (Dice = 0.92 vs 0.90), LVEF (error = 1.1% vs 3.2%), and RVEF (error = 4.05% vs 4.3%). Similarly, for native T1 maps [13]: LV myocardium (Dice = 0.88 vs 0.85), mean T1 within myocardium (R = 0.98 vs 0.82). Finally, we obtained comparable results on aortic flow with methods presented in [14] (Dice = 0.93 vs 0.94 and R = 0.89 vs 0.99, for our method and [14] respectively). It is important to note that these results were obtained on a different data set with potentially different definition of the reference standard.

Furthermore, we evaluated our cine model on the ACDC test dataset. To handle the heterogeneity of the ACDC dataset, the model was retrained on a new dataset containing both our training dataset and the ACDC training dataset. It showed results comparable to the top ten methods of the challenge. This demonstrates the ability of the model to handle a new dataset by including a small subset of images (= 200 images in this case) to its training.

Importantly, our solution is fast and fully automated, thus well-suited for use in large cohort studies. Processing the entire set of CMR sequences automatically for a single patient takes around 1 min with a GPU versus 1.5 h for manual segmentation.

## Limitations

The datasets considered for training and validation are smaller than the one used in recent works [12–14]. However, they present a very important heterogeneity in term of health conditions, and scanners. This heterogeneity is particularly important to test the robustness of the pipeline to world data. It is also interesting to note that even with small data sets, the models match the manual segmentation, especially in the case of native T1, post-contrast T1, and T2 maps where only 40 patients have been used in this study. In the case of LGE images, the Dices obtained for the LGE scar are still suboptimal, but the correlation and errors remains reasonable. Thus, we question the suitability of the Dice as a similarity measure on such small sized and fragmented structures and will consider alternative measure [15] in future iterations. Moreover, the variability of the LGE scar ground truth comes from not only the images but also from the experts, making, the problem more complicated. This might be solved in the future with a consensus on the LGE scar segmentation method [7]. Additionally, the U-Net 2D models suffer from the over-segmentation problem at the basal slice due to the variability and uncertainty among observers. Random forests models were used and have demonstrated their ability to discard the over-segmentation and improve significantly the overall results. The basal slice presents also difficulties to the deep learning algorithm in the case of the RV cavity. The results on this slice are suboptimal when compared with human contouring.

This pipeline has been furthermore applied on two independent clinical studies to evaluate the confidence one can have on the fully automatic process and decide whether or not manual intervention is required to correct the pipeline results. As shown in the results, high correlations were obtained between the fully automatic method and the corrected measures. The worst cases that had to be corrected were due to very suboptimal image quality that may also lead to human error. Moreover, these two studies were made of a variability of images coming from different scanners, hospitals, 1.5 T and 3 T, over the course of a decade. As these images were not part of the training set, we can conclude that the pipeline is already applicable to unseen data.

## Future work

Future work will tackle the issue of detecting segmentation errors within an image stack, by estimating the uncertainty of the segmentation as seen for example in [16], and by detecting outliers within the physiological parameters extracted for a study containing a large patient cohort. This work will eventually guarantee the quality of the pipeline by detecting errors and manually correcting them, thus, providing statistically accurate results, while reducing considerably the time needed to process data. More iterations are still needed to guarantee the models robustness to the variability of world data. Moreover, a significant advantage of deep learning models, is that they can learn from any data set and adapt their output automatically. This means that the pipeline could be trained with a ground truth that follows certain segmentation conventions that suits the need of a particular study. Finally, to bridge the gap between academic research and usability of algorithms, we also developed a software that allows visualization and manual alterations of the results of the deep learning segmentations if desired. This software is already used daily in our imaging research center and with collaborative teams and will hopefully be further validated on more extensive studies worldwide.

## Conclusion

The proposed pipeline allows for a fast and robust analysis of large CMR studies while also providing reproducibility. We believe that this will improve patient’s diagnosis as well as clinical studies outcome.

## Data Availability

Not applicable.

## Abbreviations

CMR: Cardiovascular magnetic resonance
EF: Ejection fraction
ED: End-diastole
EF: Ejection fraction
ES: End-systole
LGE: Late gadolinium enhancement
LV: Left ventricle/left ventricular
LVEF: Left ventricular ejection fraction
MSD: Mean surface distance
RF: Random Forest
RV: Right ventricle/right ventricular
RVEF: Right ventricular ejection fraction
